# Associations between psychological capital, risk tolerance, and satisfaction: a study of Swedish solo self-employed workers

**DOI:** 10.3389/fpsyg.2026.1780099

**Published:** 2026-04-16

**Authors:** Hampus Fougner, Claudia Bernhard-Oettel

**Affiliations:** Section for Work and Organizational Psychology, Department of Psychology, Stockholm University, Stockholm, Sweden

**Keywords:** business owners, employed gig work, job satisfaction, life satisfaction, psychological capital, risk tolerance, solo self-employment

## Abstract

**Introduction:**

Solo self-employment is a non-standard work arrangement that can be organized in various ways, such as independent business ownership or gig-by-gig employment through umbrella companies. As this sector expands, understanding the personal resources linked to worker wellbeing is essential. This study utilizes the Conservation of Resources (COR) framework to examine how personal resources regarding psychological capital (hope, self-efficacy, resilience, optimism) and risk tolerance relate to job and life satisfaction.

**Methods:**

The study analyzed cross-sectional survey data from 775 Swedish workers, distinguishing between independent business owners (*N* = 510) and umbrella-contracted gig workers (*N* = 265). Multi-group structural equation modelling was employed to compare how these personal resources relate to satisfaction outcomes across the two distinct employment forms.

**Results:**

Results revealed that the relationships with job and life satisfaction were largely consistent across groups. Future-oriented resources, specifically hope and optimism, had the strongest positive associations with both job and life satisfaction. In contrast, self-efficacy and resilience played minor roles when modeled alongside other resources. Furthermore, although business owners reported higher risk tolerance, this attitude was negatively related to life satisfaction, and no significant non-linear (inverted-U) association was found.

**Discussion:**

Despite the operational and legal differences between the studied groups, more similarities than differences were found in how personal resources relate to satisfaction outcomes. This suggests that the psychological mechanisms driving wellbeing remain relatively stable across different forms of solo self-employment, regardless of the specific employment form.

## Introduction

Self-employment—often associated with greater autonomy, adaptability, and freedom—is commonly perceived as a route to higher job and life satisfaction (De Jager et al., 2016; Van Der Zwan and Hessels, 2019). In Sweden, nearly half of the working-age population reports openness to entrepreneurial activity (Tillväxtverket, 2016), and 9.6% of the workforce is self-employed, with three-quarters of these operating without employees (Ekonomifakta, 2025). This is in line with a global trend; solo self-employment has been increasing in the workforce in most developed countries in recent decades (Cieślik et al., 2025). The self-employed without employees have also been referred to as own-account workers, which includes freelancers (van der Zwan et al., 2020).

While most individuals enter self-employment via business ownership, a growing minority work through umbrella companies. In Sweden, these umbrella companies serve as legal employers for these workers during their client assignments and handle administrative tasks (invoicing, tax payments) (Hedenus and Nergaard, 2021). However, it remains the worker’s responsibility to secure their clients and assignments. This means that these workers are employees in a legal sense, but organize their work as a self-employed worker in a practical sense (Müller et al., 2025). Hence, these two forms of solo self-employment (SSE) share similarities in how client-based work is lined up and paid for, but differ significantly in terms of legal status, risk exposure, and administrative requirements. Despite their apparent similarities (Watson et al., 2021), however, these two groups of solo self-employed workers have rarely been studied together, or compared to each other.

Although research on self-employment and wellbeing has received increasing attention (Stephan, 2018), the wealth of studies typically concentrates on business owners and rarely differentiates between those with and without employees (Van Der Zwan and Hessels, 2019). Seen as a whole group, self-employed business owners often report higher job satisfaction than wage earners, but findings for life satisfaction are somewhat mixed (Binder and Blankenberg, 2021). These findings have mainly been explained with the specific employment and working conditions in self-employment: the relatively high levels of job autonomy and flexibility in self-employment may predominantly boost job satisfaction (Harrop et al., 2025; Lee and Bae, 2024) while on the other hand, income volatility, complex regulations, and blurred work–life boundaries may erode satisfaction with life in self-employment (Stephan, 2018). One of the few studies that differentiated between solo self-employment and businesses with employees (van der Zwan et al., 2020) found that the solo self-employed reported equal or higher satisfaction in certain life domains when compared to business owners with employees. An explanation for this finding is that business owners with employees may, on the one hand, experience higher pressure and demands since they need to coordinate employees, while, on the other hand, they also have greater decision autonomy over their work, since they can delegate tasks (Prottas and Thompson, 2006). However, while the role of job characteristics and their link to job and life satisfaction has received considerable research attention, individual differences, known to predict (solo) self-employment preferences (e.g., Baluku et al., 2021; Howard and Boudreaux, 2024; Karlsson Linnér et al., 2019; Kritikos, 2022), remain underexplored in research on self-employment. These differences, however, may be valuable resources, which potentially fuel job and life satisfaction. Another limitation of earlier research lies in the fact that solo self-employed workers without registered businesses (who are employed by umbrella companies) have rarely been studied (Hedenus and Nergaard, 2021) so that little is known about job and life satisfaction in this type of SSE.

Addressing these voids, the aim of this study is threefold. First, based on the conversation of resources (COR) theory, we focus on the role of individual differences in terms of psychological capital to study job and life satisfaction in SSE. Second, we focus on the role of risk tolerance as an individual difference among the self-employed. Although risk tolerance may be of importance in self-employment (Howard and Boudreaux, 2024), its role for job and life satisfaction is largely unknown, so this study will contribute with relevant new knowledge. Third and finally, we also explore whether psychological capital and risk tolerance relate to job and life satisfaction in similar ways in the solo self-employed with a registered business, and those employed by umbrella companies. Although the way of acquiring and working for clients is fairly similar in both types of solo self-employments, legal differences may lead to differences in the amount of responsibility and autonomy, as well as the importance (and possibility) of investments. However, whether or not this means that the role of psychological capital or risk tolerance differs for job and life satisfaction in solo business owners or those employed through umbrella companies has rarely been studied before. By exploring this question, our study can help to understand whether certain personal resources are more beneficial in specific contexts or if they play a similar role regardless of the exact form of SSE. This insight is important for both theoretical and practical reasons. Differentiating among forms of solo self-employment advances theory development and research quality since it may inform the design of future research studies. For practitioners and institutions supporting self-employed individuals, it may be advantageous to understand how individual differences relate to job and life satisfaction, and whether the context of solo self-employment (own business versus employment through umbrella company) makes a difference.

### A conservation-of-resources perspective

Conservation of Resources (COR) theory (Hobfoll, 1989) proposes that individuals are motivated to obtain, retain, protect, and grow valued resources. These resources—ranging from material assets and social conditions to skills, energies, and psychological characteristics—form the foundation from which people pursue goals, manage stress, and maintain wellbeing. According to COR, stress arises when resources are threatened, lost, or when individuals fail to gain expected returns on resource investments. Importantly, resource loss is more potent and harmful than resource gain, often triggering downward loss spirals, whereas accumulated resources can initiate upward gain spirals (Hobfoll, 2002).

A central premise of COR theory is that resources rarely operate in isolation: they cluster and build upon one another over time, creating “resource caravans” that can either stabilize wellbeing or exacerbate vulnerability. Solo self-employment (SSE), with its characteristic mix of autonomy, financial unpredictability, and blurred work–life boundaries, represents a particularly volatile “caravan passageway” (Le et al., 2014; Wheeler et al., 2012). Within this passageway, individuals face both heightened risks of resource depletion, such as income insecurity or work overload, and opportunities for substantial resource gains, such as expanded skills, market opportunities, and identity enrichment.

In this context, psychological capital (PsyCap) and risk tolerance emerge as especially valuable personal resources for solo self-employed individuals. PsyCap—comprising hope, efficacy, resilience, and optimism—provides a multifaceted reservoir that supports adaptive action, perseverance, and recovery in the face of setbacks. Each component can initiate gain spirals by fostering confidence, goal pursuit, and constructive interpretation of challenges (Luthans et al., 2007). Likewise, risk tolerance can serve as a catalytic resource: it helps individuals engage effectively with uncertainty, make strategic decisions under ambiguity, and translate the inherent risks of SSE into opportunities for growth rather than threats of loss (Howard and Boudreaux, 2024).

By integrating these personal resources into a COR framework, we argue that individuals with higher PsyCap and risk tolerance are better equipped to navigate the instability of SSE. These resources not only buffer against potential loss spirals but also increase the likelihood of generating resource gains that enhance both job and life satisfaction. Thus, examining PsyCap and risk tolerance offers a more nuanced understanding of how personal resource reservoirs shape sustainable and satisfying solo self-employment careers.

### Psychological capital as a key resource

Psychological Capital (PsyCap) integrates four malleable personal resources, namely the “HERO” dimensions of hope (self-)efficacy, resilience, and optimism (Luthans et al., 2007). Hope is characterized by both the motivation and ability to identify alternative pathways to reach desired outcomes (Snyder et al., 1996). Self-efficacy reflects an individual’s confidence in their ability to successfully tackle challenges and achieve goals (Bandura, 1997). Resilience involves the capacity to recover from setbacks and leverage these experiences as opportunities for growth and development (Luthans et al., 2007). Optimism represents positive attributions and a belief in future success. Together, these four personal resources promote goal pursuit, adaptability, and positive expectations; and they have been linked to numerous outcomes: job satisfaction, life satisfaction, engagement, commitment, performance, creativity, meaningfulness, lower burnout, and reduced cynicism (Avey et al., 2011; Kong et al., 2018; Liu et al., 2025; Wu and Nguyen, 2019). Longitudinal research further shows associations with higher daily positive mood (Culbertson et al., 2010) and reduced stress during crises (Pan et al., 2024). In self-employment, in which individuals more directly “own” the outcomes of their actions, PsyCap may be particularly valuable. For example, Baron et al. (2016) found that self-employed workers reported lower stress than other occupational groups, partly due to higher PsyCap. PsyCap has also been linked to the self-employed’s ability to navigate uncertainty, secure resources, build teams, and adapt in dynamic environments (Bass et al., 2024; Hmieleski et al., 2015). Although little research has examined PsyCap in SSE, its relevance for resilience, proactivity, and adaptability suggests it could be a critical resource for upholding satisfaction levels in non-standard forms of work (Ashford et al., 2018). Therefore, our first hypothesis (H1) reads:

*Hypothesis 1*: Among SSE workers in Sweden, the four PsyCap dimensions—self-efficacy, optimism, resilience, and hope—are positively associated with (a) job satisfaction and (b) life satisfaction.^1^

### Risk tolerance—resource or risk?

Beyond personal resources, personal dispositions toward uncertainty may also shape satisfaction in SSE. Risk tolerance—the degree to which individuals accept variability in outcomes—has shown curvilinear links with performance and satisfaction. For the self-employed and perhaps in particular those with entrepreneurial ambitions to grow their business, moderate risk tolerance is associated with greater business survival compared to very low or very high tolerance (Caliendo et al., 2010, 2014). This may be explained by the fact that an underestimation of risks (Bandura, 2009; Dohmen et al., 2023; Kiegler et al., 2022; Tipu, 2017), may result in poor financial investments among entrepreneurs (Díaz-Pincheira et al., 2025), whereas an overestimation may be associated with missing out on good business opportunities. A moderate preference for risk among successful entrepreneurs may also be explained in a COR perspective: entrepreneurs often have to manage risk very carefully to preserve the limited resources of their business in order to – over time – accumulate capital through profit. Being able to take risks at a moderate level may thus also relate positively to job and life satisfaction, since the entrepreneur, or self-employed individual, should feel a sense of accomplishment and contentment when their business goes well. An inverted-U pattern has likewise been found for risk taking propensity in a more general sense and life satisfaction in the general population (Thunström et al., 2025). Taken together, earlier research points to beneficial effects of moderate risk taking in self-employment, and a link of moderate risk taking and satisfaction in the general population. Accordingly, our second hypothesis reads:

*Hypothesis 2*: Risk tolerance shows an inverted-U-shaped relationship with (a) job satisfaction and (b) life satisfaction among SSE workers.

### The type of SSE: exploring potential differences

Solo self-employment encompasses a wide variety of work arrangements (Watson et al., 2021) and personal motivations (van den Groenendaal et al., 2021), making it a highly heterogeneous employment form. In Sweden, two prominent forms are SSE business owners, who register and operate their enterprises independently, and SSE non-business owners, who work through umbrella companies. Business owners maintain business status continuously, manage all operations, bear full administrative and financial responsibility, and enjoy greater entrepreneurial autonomy and potential tax benefits. Having registered a business also means being able to make business investments (e.g., for buying equipment) that can be deducted from the annual tax declaration. This opportunity is nonexistent for those who work for a client via an umbrella company. These non-business owners outsource invoicing of clients to umbrella companies, thereby reducing administrative load but also limiting financial autonomy and entrepreneurial development (Hedenus and Nergaard, 2021). For the time of their work with the client, these workers are formally employed as short-term temporary workers with the umbrella company as their employer. This means that their employer takes contact with clients and enforces payment, deducts taxes and fees and pays a salary to the workers (Bernhard-Oettel et al., 2025). This type of SSE work may attract individuals who feel less secure about their abilities to put up with difficult clients or complicated tax regulations, as well as it may mean that risk tolerance, particularly in regard to business investments are of less importance to thrive. In other words, these structural distinctions may shape both the levels of—and the ways in which—PsyCap and risk tolerance relate to satisfaction. For example, it is conceivable that resilience is important for job and life satisfaction in both business and non-business owners, but that the relationship is significantly stronger in business owners who have no employer or organization to turn to when clients do not pay or when tax regulations are difficult to understand. It may also be the case that risk tolerance at a moderate level is significantly and positively related to job and life satisfaction in business owners, whereas perhaps no such relationship does exist in non-business owners. However, since research in self-employment typically does not disentangle different types or legal forms of self-employment, a detailed, more directed hypothesis is hampered and we therefore take an exploratory approach by formulating the following set of research questions (RQ1, RQ2):

*Research Question 1 (RQ1)*: Do SSE business owners and non-business owners in Sweden differ in their mean levels of PsyCap dimensions and risk tolerance?

*Research Question 2 (RQ2)*: Do the associations between PsyCap dimensions, risk tolerance, and satisfaction differ between SSE business owners and non-business owners in Sweden?

## Materials and methods

### Procedure

A cross-sectional survey design was used, with data collected between late autumn 2021 and spring 2022. SSE business owners were recruited via newsletters and mailings from relevant professional associations, while SSE non-business owners were recruited through their respective umbrella companies. Additional participants were reached via social media, postal invitations distributed by Statistics Sweden (SCB), and snowball sampling, to capture the diversity of the SSE population in Sweden.

Participants accessed the secure online survey, received written information on the study’s purpose, data handling, and voluntary nature of participation, and provided active informed consent. Eligibility was confirmed through screening questions requiring participants to have earned income within the past 12 months from either (a) self-owned business operations without employees or (b) umbrella company–based self-employment. The study was approved by the Swedish Ethical Review Authority (Dnr: 2021–03852; 2022–00626-02; 2022–01742-02).

### Participants

Of the 1,278 individuals who accessed the survey, 503 exited without answering any questions or did not meet eligibility criteria, leaving a final sample of 775 SSE workers: 510 business owners and 265 non-business owners. Overall, 56.7% of participants identified as women, 43.3% as men, and 10 selected “other/do not want to answer,” which was coded as missing for gender-based analyses due to the small group size. Ages ranged from 21 to 87 years (M = 53.05, SD = 12.20), and 53.1% held a bachelor’s degree or higher. The largest occupational sectors were culture and entertainment (19.1%), communication and information (15.6%), and education (9.1%), with others represented across construction, healthcare, and various business services. Most participants resided in large cities, and 89.8% were born in Sweden. A comparison of the two groups’ profiles is provided in Table 1. Briefly, business owners reported longer tenure in their current SSE form, higher levels of income, and a higher proportion of women among business owners compared to non-business owners.

**Table 1:** Profile of study participants across groups.

Variable	Business owners (*N =* 440–510)	Non-business owners (*N =* 213–263)
Age (years)		
Mean (*SD*)	53.25 (11.63)	52.64 (13.49)
Range (min–max)	22–87	21–80
Employment-form tenure (years)		
Mean (*SD*)	12.36 (10.62)	5.26 (6.40)
Gender (%)		
Women	63.0	43.8
Men	37.0	56.2
Education (%)		
Bachelor’s degree or higher	56.0	46.0
Income from SSE (%)		
Entire income derived from SSE	74.9	43.2
Yearly income over 250,000 SEK	67.6	28.5

### Measures

#### Satisfaction

Job satisfaction was assessed with the item: “I am content with the job I have.” (Brayfield and Rothe, 1951), and life satisfaction with the item: “I am satisfied with my life” (Diener et al., 1985; Hultell and Gustavsson, 2008). Both items were rated on a 5-point Likert scale ranging from 1 (“strongly disagree”) to 5 (“strongly agree”). Such single-item measures have demonstrated good levels of both validity and reliability for (multiple) global constructs targeting job and life satisfaction in psychological research (Fisher et al., 2016; Jovanović and Lazić, 2020; Nagy, 2002).

#### Risk tolerance

To assess individual differences in risk tolerance, participants responded to a hypothetical lottery investment scenario inspired by Block et al. (2015) and Caliendo et al. (2010), comparable to the question used in the German Socio-Economic Panel Survey (SOEP).

Risk tolerance refers to an individual’s attitude towards financial risk. Participants were asked: “Imagine you have won 1 million SEK in a lottery. After having received the money, you have the possibility to invest the money in an entrepreneurial activity. With a probability of 50%, you double the amount. With a probability of 50%, you would lose half of the invested money. How much money obtained from the lottery would you invest?”. The participants could choose to invest between 0 and 1 million SEK in intervals of 100,000 SEK.

Propensity-based measures, such as this, are suitable for large-scale surveys and capture domain-relevant psychological constructs (Bagaïni et al., 2025), are widely used in the psychology of behavioral economics (e.g., Kahneman and Tversky, 1979). Despite being hypothetical, the question has been shown to differentiate between self-employed subgroups, such as necessity- and opportunity-driven entrepreneurs (Block et al., 2015) and to predict entrepreneurial success, entry, and turnover (Caliendo et al., 2010, 2014).

#### Psychological capital

Self-efficacy (Parker, 1998), optimism (Scheier and Carver, 1985), hope (Snyder et al., 1996), and resilience (Wagnild and Young, 1993) were measured using the psychological capital questionnaire (PCQ; Luthans et al., 2007), using a six-point Likert scale ranging from 1 (“strongly disagree) to 6 (“strongly agree”). A sample item is: “I feel confident analyzing a long-term problem to find a solution” (self-efficacy; Luthans et al., 2007)^2^.

To minimize respondent burden and increase completion rates, a reduced version of the scale was used. 14 out of 24 items were carefully selected to ensure clarity and relevance for the target population while preserving the scale’s psychometric integrity. Self-efficacy, optimism, and resilience consisted of 4 items each. Two items were selected for measuring hope. Confirmatory Factor Analyses (CFAs) were conducted to assess the scale’s structure for both business owners (*N =* 451) and non-business owners (*N =* 225). The results suggested that a four-factor structure was a better fit to the data, in both groups, than a single-factor and a second-order structure (see Supplementary material). A bifactor model was also tested, but it did not converge. Therefore, we also assessed discriminant validity (whether the scales were empirically distinct) following the recommendations by Rönkkö and Cho (2022). Factor covariances ranged between 0.48 and 0.73 across groups, with upper 95% confidence intervals being < 0.83. While this suggested a high degree of correlation between the constructs, it does not necessitate a termination of further analyses. Furthermore, the scales showed good internal consistency in both samples. McDonald’s omega coefficients ranged from 0.77 to 0.86 across all four sub-scales (optimism, resilience, hope, and self-efficacy).

#### Control variables

Based on the results in prior studies (Avey, 2014; Block et al., 2015; Bockorny and Youssef-Morgan, 2019; Fakunmoju, 2020; König, 2021; VanderWeele et al., 2025), age (in years), gender (1 = men), tenure in SSE (in years), and education (1 = ≥B. A. degree/academic education) were included in this study.

### Analytic approach

Initial data screening showed some skewed data distributions across the study variables (< |1.87| across groups), and the resilience items generally showed a high kurtosis across both groups (range: 0.08 to 4.16). Approximately 12% of the data were missing, these were highest for risk tolerance (~16%) and the PsyCap items (~14%). Furthermore, we corrected ten implausible outlier responses for age and SSE-tenure (in years) and normalized the risk tolerance variable from its original range to a 0–1 scale for interpretability. Demographic variables such as gender and education were coded as dichotomous variables. Observed scale means were calculated when at least two items were present, or a single item score was used to represent the scale mean.

Given the data characteristics, structural equation modeling (SEM) was conducted in R using the lavaan package (Rosseel, 2012) with robust maximum likelihood estimation (MLR) and FIML for missing data. Single-indicator latent variables were specified with fixed loadings and residuals. Model fit was evaluated with scaled *χ*^2^, df, and robust indices. Conventional fit indices were used to evaluate model fit (Hu and Bentler, 1999), suggesting a Mean Square Error of Approximation (RMSEA < 0.06: very good fit), the Standardized Root Mean Square Residual (SRMR < 0.06: good fit), and the Comparative Fit Index (CFI < 0.95: good fit). An RMSEA/SRMR ≤ 0.08 and CFI ≥ 0.90 indicate a moderate fit. However, values were interpreted as guidelines and not dichotomous cut-off points, taking into account the data quality, design, and prior knowledge (Amrhein et al., 2019; Fife et al., 2022).

Measurement invariance across solo self-employed business owners and non-owners was tested via multi-group CFA (Somaraju et al., 2022; Vandenberg and Lance, 2000). Three models were compared: (1) a configural model, which allowed all parameters to vary freely across groups, (2) a metric model with constrained intercepts, and (3) a scalar model with constrained intercepts and factor loadings. Following the recommendations by Somaraju et al. (2022) these models were compared using scaled *χ*^2^ difference tests alongside ΔCFI, ΔRMSEA, and ΔSRMR, with ΔCFI ≤ 0.002 as the primary criterion (Meade et al., 2008). To assess the magnitude of measurement non-equivalence, we computed d_MACS_ (Differential item functioning Magnitude and Common factor Standardized, see Nye et al., 2019; Nye and Drasgow, 2011). In essence, this provides an effect size measure that can be interpreted like Cohen’s *d*, with values of 0.4, 0.6, and 0.8 considered as a small, medium, or large effect size (Nye et al., 2019). Thereby, these statistics reflect a degree of uncertainty (predicted bias) in the estimated parameters (Robitzsch and Lüdtke, 2023).

Latent means were compared by constraining the mean of the latent factor to zero in a reference group while allowing it to be freely estimated in the other group. The estimated mean parameter in the second group thus represents the average difference in latent means between the groups. Hypotheses were tested using multi-group SEM (latent regression), including job and life satisfaction as outcomes. Curvilinear statistical effects of risk tolerance were modeled using the double-mean-centering approach (*modsem*; Sluppaug et al., 2024). To explore whether group membership could moderate the relationships (RQ2), we tested for relational invariance by constraining the structural paths across groups (Somaraju et al., 2022). Given the high intercorrelations among the PsyCap scales, unique contributions of predictors were assessed using ΔR^2^ based on model-implied correlations (Hayes, 2021). Group-specific predictions were visualized with empirical Bayes latent scores (Fife et al., 2022).

Age, gender, tenure, and education were initially included as covariates. However, since the age variable showed the highest influence on the model estimates, the interpretation of the results remained consistent when age was considered the sole covariate. Therefore, the reduced model was used to maximize the number of participants included in the analysis. Likewise, risk tolerance showed a negligible impact on other variables’ coefficients.

## Results

### Descriptive statistics

Table 2 presents observed means, standard deviations, and bivariate correlations of the study variables separately for SSE business owners (*N =* 440–509) and non-business owners (*N =* 213–263).

**Table 2:** Means, standard deviations, and bivariate correlations (Pearson’s *r*) for business owners (*N =* 440–509; lower matrix) and non-business owners (*N =* 213–263; upper matrix).

	Business owners		Non-business owners		1	2	3	4	5	6	7	8	9	10	11
	Mean	*SD*	Mean	*SD*											
1. Gender [Men]					-	−0.23***	0.09	0.12	0.11	0.07	0.01	0.07	−0.01	0.05	0.03
2. Education [High]					−0.17***	-	−0.07	−0.09	−0.01	−0.19**	−0.10	−0.04	−0.01	−0.15*	0.04
3. Age	53.25	11.63	52.64	13.49	0.12**	−10*	-	0.11	0.19**	0.35***	0.17*	0.18**	−0.26***	0.20**	0.29***
4. Tenure	12.40	10.62	5.24	6.37	0.13**	−0.19***	0.54***	-	−0.01	−0.00	0.04	−0.10	−0.09	−0.01	−0.09
5. Self-Efficacy	4.94	0.84	4.90	0.90	−0.01	0.04	0.17***	0.04	-	0.53***	0.61***	0.61***	−0.06	0.38***	0.45***
6. Optimism	4.36	0.98	4.39	1.08	−0.02	−0.09	0.29***	0.15**	0.56***	-	0.47***	0.54***	−0.10	0.48***	0.58***
7. Resilience	5.05	0.73	5.13	0.75	−0.01	−0.01	0.12*	0.10*	0.58***	0.54***	-	0.48***	−0.11	0.33***	0.33***
8. Hope	4.42	1.15	4.54	1.22	0.13**	−0.02	0.18***	0.08	0.56***	0.53***	0.46***	-	−0.11	0.50***	0.63***
9. Risk Tolerance	0.38	0.25	0.27	0.22	0.07	−0.02	−0.02	0.04	0.10*	0.11*	−0.01	0.04	-	−0.07	−0.07
10. Job Satisfaction	4.13	1.01	4.03	1.17	−0.00	−0.10*	0.20***	0.11*	0.28***	0.40***	0.33***	0.48***	−0.02	-	0.44***
11. Life Satisfaction	3.96	0.90	3.96	0.97	0.05	−0.02	0.19***	0.09	0.35***	0.50***	0.29***	0.47***	−0.05	0.41***	-

Correlation coefficients for SSE business owners are presented in the lower diagonal, and non-business owners in the upper diagonal. Coding: Gender (0 for women, 1 for men); Education (0 < B. A. degree, 1 ≥ B. A. degree). *p < 0.05, **p < 0.01, ***p < 0.001.

On average, business owners reported slightly higher job satisfaction (M = 4.13, SD = 1.01) than non-business owners (M = 4.03, SD = 1.17), with the latter group displaying greater variability. Most of the correlations were statistically significant, with the exception of risk tolerance and the two outcomes. The strongest association was found between hope and life satisfaction, with slightly stronger associations among non-business owners than among business owners. A moderate positive correlation between job and life satisfaction was found in both groups.

### Measurement invariance

A series of nested models was tested using MGCFA to examine measurement invariance between SSE business owners (*N =* 468) and non-business owners (*N =* 231). The configural model showed acceptable fit (*χ*^2^ [202] = 472.103, *p* < 0.001, CFI = 0.936, RMSEA = 0.069, SRMR = 0.056), indicating that the factor structure was similar across groups. Imposing equality constraints on factor loadings across groups led to a negligible decrease in model fit (Δ*χ*^2^ [10] = 12.863, *p* = 0.231, ΔCFI = −0.001, ΔRMSEA = −0.001, ΔSRMR = 0.000), suggesting that metric invariance holds. When the scalar model was compared with the metric model, a statistically significant difference was found (Δ*χ*^2^ [10] = 23.939, *p* = 0.008, ΔCFI = −0.003, ΔRMSEA = 0.000, ΔSRMR = 0.001). However, ΔSRMR and ΔRMSEA did not indicate any major difference, and the ΔCFI showed only a minor deviation (i.e., 0.001) using strict cutoff recommendations of 0.002. In a further investigation of these differences, item-level d_MACS_ values ranged from 0.04 to 0.37. Some optimism items showed statistically significant differences across the two groups. However, the predicted bias at scale-level optimism was negligible (d_MACS_ = 0.22). This indicates that while minor item-level non-equivalence was present, these biases likely offset one another, resulting in negligible group differences in the composite scale scores. Therefore, the measurement model was left with equal constraints and passed for further analyses.

### Relationships of psychological capital with job and life satisfaction

An MGSEM was conducted to test hypotheses regarding job satisfaction (H1a) and life satisfaction (H1b) among business owners (*N =* 460) and non-business owners (*N =* 224). The fitted model was based on the previous scalar model, where the intercepts and factor loadings were set to be equal across groups. Latent variables were allowed to correlate. Age was included as a covariate. Fit indices suggested a moderate fit to the data (*χ*^2^ [256] = 642.057, *p* < 0.001, CFI = 0.910, RMSEA = 0.077, SRMR = 0.074).

*Results for job satisfaction.* Among business owners, hope (*b* = 0.44, CI95 [0.28, 0.60], *p* < 0.001, *β* = 0.49) and optimism (*b* = 0.20, CI95 [0.01, 0.38], *p* = 0.040, *β* = 0.18) showed both positive significant relationships with job satisfaction, while the other variables were held constant. Furthermore, a negative association was found between self-efficacy and job satisfaction (*b* = −0.39, CI95 [−0.67, −0.10], *p* = 0.008, *β* = −0.25). The association between resilience and job satisfaction was statistically non-significant (*b* = 0.26, CI95[−0.10, 0.62], *p* = 0.160, *β* = 0.01). Overall, the variables accounted for 31% of the variance in job satisfaction in this group. Among non-business owners, hope (*b* = 0.30, CI95 [0.04, 0.56], *p* = 0.025, *β* = 0.32) and optimism (*b* = 0.38, CI95 [0.12, 0.65], *p* = 0.004, *β* = 0.33) were most strongly related to job satisfaction, when adjusting for age and the remaining PsyCap dimensions. Neither self-efficacy nor resilience showed a notable relationship with job satisfaction. In this group, the variables explained 33% of the variance in job satisfaction.

*Results for life satisfaction.* Among business owners, both optimism (*b* = 0.41, CI95 [0.24, 0.58], *p* < 0.001, *β* = 0.42) and hope (*b* = 0.27, CI95 [0.14, 0.41], *p* < 0.001, *β* = 0.32) were positively associated with life satisfaction. No significant associations were found between self-efficacy or resilience and life satisfaction. Together, the variables explained 33% of the variance in life satisfaction. Among non-business owners, optimism showed a similar positive relationship (*b* = 0.30, CI95 [0.11, 0.49], *p* = 0.002, *β* = 0.3), while the coefficients concerning hope indicated a stronger association with life satisfaction (*b* = 0.50, CI95 [0.30, 0.69], *p* < 0.001, *β* = 0.63). Neither self-efficacy nor resilience reached statistical significance in this model. The variables explained 52% of the variance in life satisfaction among non-business owners.

Taken together, the results partially support the first hypotheses concerning job satisfaction (H1a) and life satisfaction(H1b). More specifically, the results regarding optimism and hope supported the hypotheses in both groups of SSE workers. Contrary to H1a, self-efficacy had a significant negative association with job satisfaction in business owners, no significant association with job satisfaction in non-business owners, and, rejecting H1b in this regard, no significant relation to life satisfaction in any of the groups. In addition, and also contrasting our expectations, resilience was not significantly related to job or life satisfaction.

### Risk tolerance

To evaluate potential curvilinear associations between risk tolerance and the two outcomes (H2), risk tolerance, including a double-mean-centered quadratic term (risk tolerance x risk tolerance), was fitted together with the four “HERO” variables and age. The statistical model showed a moderate fit to the data (*χ*^2^ [274] = 688.921, *p* < 0.001, CFI = 0.909, RMSEA = 0.073, SRMR = 0.075).

As presented in Tables 3 and 4, the results did not support our second hypothesis; none of the quadratic terms for risk tolerance showed statistically significant relations with either outcome. Instead, the results indicated that risk tolerance was weakly, but negatively related to life satisfaction among business owners (*b* = −0.38 CI95[−0.70, −0.05], *p* = 0.023, *β* = −0.10), while adjusting for PsyCap (see Supplementary material for further analyses). However, the variable did not strongly contribute to the explained variance in life satisfaction (Δ*R*2 = 0.01). No further support for the second hypothesis was found across groups and outcomes. This means that H2 was rejected.

**Table 3:** Latent regression coefficients across groups with job satisfaction as an outcome (MGSEM).

	Business owners (*N =* 463)				Non-business owners (*N =* 230)			
	*b*	(CI95)	*β*	Δ*R*^2^	*b*	(CI95)	*β*	Δ*R*^2^
Hope	0.44***	(0.27, 0.60)	0.49	0.12	0.30*	(0.04, 0.57)	0.32	0.04
Self-Efficacy	−0.37*	(−0.65, −0.09)	−0.24	0.02	−0.03	(−0.53, 0.48)	−0.01	0.00
Resilience	0.24	(−0.13, 0.61)	0.12	0.01	0.00	(−0.59, 0.58)	0.00	0.00
Optimism	0.20*	(0.01, 0.39)	0.18	0.01	0.39**	(0.12, 0.65)	0.33	0.05
Risk Tolerance	−0.18	(−0.58, 0.22)	−0.04	0.00	0.10	(−0.59, 0.80)	0.02	0.00
Risk Tolerance^2^	0.35	(−0.73, 1.43)	0.03	0.00	−1.71	(−3.86, 0.65)	−0.10	0.01
Age	0.01*	(0.00, 0.01)	0.01	0.01	0.00	(−0.01, 0.01)	0.05	0.00
*R^2^*	0.31				0.34			

CI95 represents the 95% confidence interval for the unstandardized regression coefficient (b). *β* = Standardized regression coefficient. Model fit: *χ*^2^ (274) = 688.921, *p* < 0.001, CFI = 0.909, RMSEA = 0.073, SRMR = 0.075. *p < 0.05, **p < 0.01, ***p < 0.001.

**Table 4:** Latent regression coefficients across groups with life Satisfaction as an outcome (MGSEM).

	Business owners (*N =* 463)				Non-business owners (*N =* 230)			
	*b*	(CI95)	*β*	Δ*R*^2^	*b*	(CI95)	*β*	Δ*R*^2^
Hope	0.26***	(0.13, 0.39)	0.33	0.05	0.51***	(0.31, 0.71)	0.64	0.16
Self-Efficacy	−0.07	(−0.32, 0.18)	−0.05	0.00	−0.27	(−0.72, 0.18)	−0.20	0.01
Resilience	−0.22	(−0.55, 0.11)	0.12	0.01	−0.06	(−0.47, 0.35)	0.03	0.00
Optimism	0.44***	(0.26, 0.61)	0.44	0.08	0.30**	(0.11, 0.49)	0.31	0.05
Risk Tolerance	−0.38*	(−0.70, −0.05)	−0.10	0.01	0.19	(−0.33, 0.71)	0.04	0.00
Risk Tolerance^2	−0.04	(−0.90, 0.83)	0.00	0.00	0.44	(−1.14, 2.02)	0.03	0.00
Age	0.00	(−0.00, 0.01)	0.04	0.00	0.01	(0.00, 0.02)	0.11	0.01
*R^2^*	0.34				0.52			

CI95 represents the 95% confidence interval for the unstandardized regression coefficient (b). *β* = Standardized regression coefficient. Model fit: *χ*^2^ (274) = 688.921, p < 0.001, CFI = 0.909, RMSEA = 0.073, SRMR = 0.075. *p < 0.05, **p < 0.01, ***p < 0.001.

### The type of solo self-employment: exploring group differences

To answer RQ1, latent mean differences were examined between solo self-employed business owners and non-business owners. Group means were estimated relative to business owners (set to zero for identification). Results suggested that non-business owners, on average, reported significantly lower risk tolerance (*Est*. = − 0.10, CI95 [−0.14, −0.06], *p* < 0.001, *SD* = −0.41), corresponding to approximately 100,000 SEK less invested in the hypothetical lottery question. No statistically significant latent mean differences emerged for self-efficacy (*Est*. = − 0.04, CI95 [−0.15, 0.08], *p* = 0.547), optimism (*Est*. = 0.06, CI95 [−0.11, 0.23], *p* = 0.479), resilience (*Est*. = 0.07, CI95 [−0.03, 0.16], *p* = 0.170), or hope (*Est*. = 0.13, CI95 [−0.07, 0.32], *p* = 0.214). Overall, these results suggest that the two groups shared a similar psychological profile, differing primarily in their level of risk tolerance.

**Figure 1:**
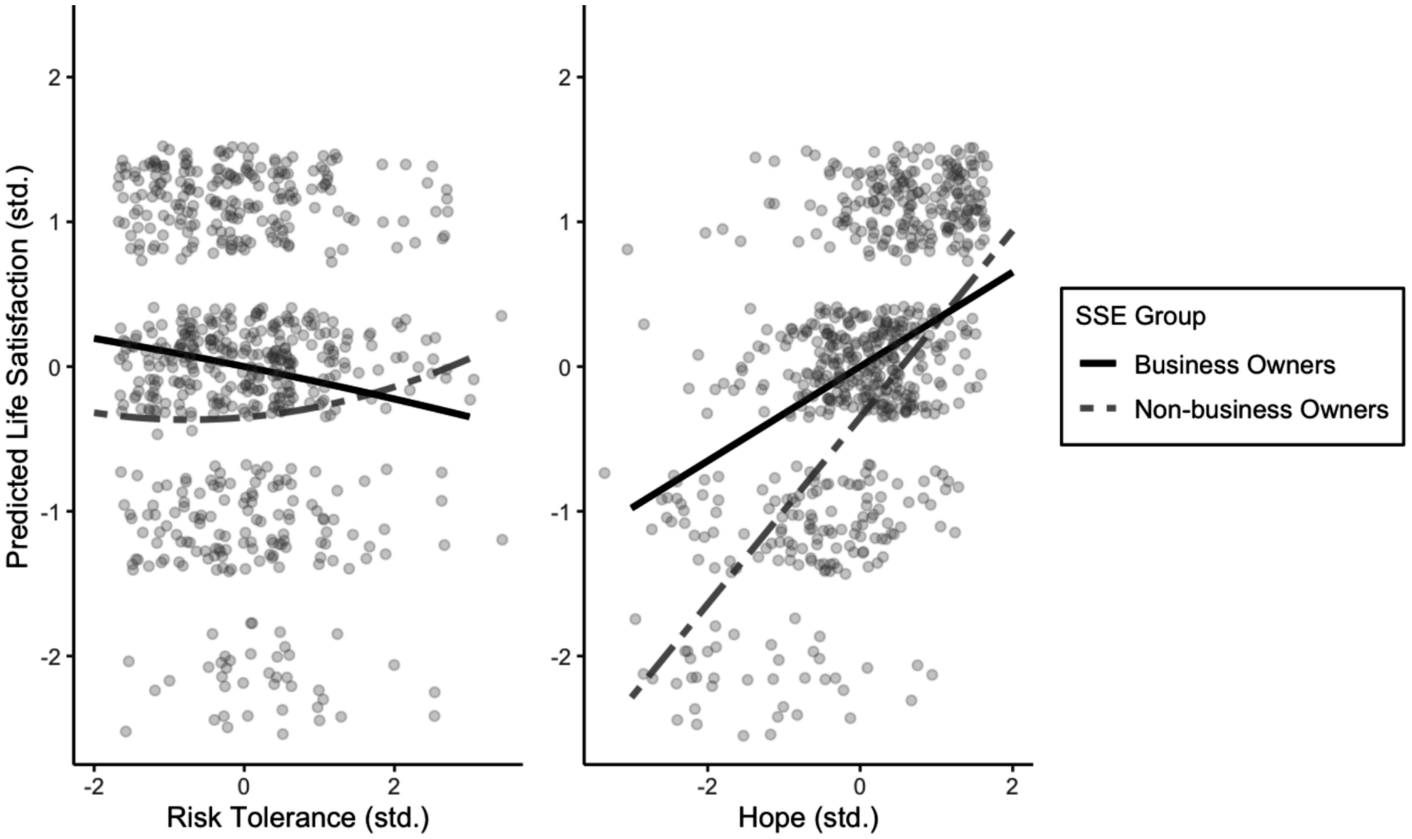
Model-estimated life satisfaction depending on (curvilinear) risk tolerance and hope for each group. Life satisfaction was plotted across standardized values of curvilinear risk tolerance and hope based on standardized regression coefficients from the multi-group SEM model. Predictions were estimated at mean age and PsyCap. Empirical Bayes scores were overlaid as points.

To explore whether the type of SSE could moderate the relationships with job and life satisfaction (RQ2), a series of nested model comparisons was conducted individually for each of the regression paths. When the relations to life satisfaction were constrained across groups for risk tolerance (*χ*^2^ [1] = 4.025, *p* = 0.045, ΔCFI = 0, ΔRMSEA = 0, ΔSRMR = 0) and hope (*χ*^2^ [1] = 5.266, *p* = 0.022, ΔCFI = −0.001, ΔRMSEA = 0, ΔSRMR = 0), the results indicated a statistically significant difference, yet a minimal decrease in model fit. To better understand these group differences, Figure 1 illustrates how the associations of risk tolerance (left panel) and hope (right panel) with life satisfaction differed between business owners and non-business owners. Among business owners, higher risk tolerance was associated with lower life satisfaction. In contrast, for non-business owners the association between risk tolerance and life satisfaction was not significant, and the confidence interval included zero. This indicates that the negative association between risk tolerance and life satisfaction was specific to the business owner group, whereas no such link was observed for non-business owners. Hope, showed the opposite pattern: In both groups, increases in hope related to increases in life satisfaction. However, the slopes differ in steepness between the two groups, and as can be seen, hope was more strongly related to life satisfaction among non-business owners than among business owners.

All other paths were invariant (ΔCFI = 0, ΔRMSEA = 0, ΔSRMR = 0) and statistically non-significant. In sum, the relationship between all tested predictors and job satisfaction was similar for both groups. For life satisfaction, the above-described differences were found for hope and risk tolerance, whereas the relationship to resilience, optimism and self-efficacy did not differ between the two groups. Despite these nuances in life satisfaction, the results therefore suggests that the two groups appear functionally similar in how their personal resources relate to job satisfaction.

## Discussion

This study examined how psychological capital (the four HERO dimensions) and risk tolerance were related to job and life satisfaction among solo self-employed workers in Sweden, comparing business owners and umbrella-contracted non-owners.

Consistent with prior research (Avey et al., 2011; Nolzen, 2018), all four of the HERO dimensions showed positive correlations with job and life satisfaction. However, when controlling for age and risk tolerance, hope and optimism emerged as the strongest statistical predictors of both job and life satisfaction, supporting the evidence that future-oriented cognitions are central to overall wellbeing (Gallagher and Lopez, 2009; VanderWeele et al., 2025). In contrast, self-efficacy and resilience explained little unique variance once other resources were modeled. Furthermore, although there are suggestions that high-efficacy workers may set demanding internal performance benchmarks, where any shortfall against these self-referent criteria could sharpen dissatisfaction (e.g., Bandura, 1997), the statistically significant negative coefficient for self-efficacy should be interpreted cautiously, likely reflecting suppression effects rather than a substantive relationship. Overall, these findings align with the Conservation of Resources theory, where prospective cognitions align with wellbeing (Hobfoll, 1989). Whether the relationship between these personal resources and wellbeing stabilizes over time and buffers against loss spirals as theoretically expected (Hobfoll, 2002; Kačmár et al., 2024) would be an important question for future research.

Regarding the moderating role of the SSE context, few differences between business owners and non-business owners were found in how psychological capital was related to job and life satisfaction. This means that by and large, these psychological capital factors associate in similar ways with job and life satisfaction of SSE business owners and non-owners. This shared psychological reality aligns with the practical similarities in how client-based work is organized and executed across both groups (Müller et al., 2025). One exception was found for the factor hope: hope was more strongly related to life satisfaction among non-owners. An explanation for this finding could be that umbrella-contracted SSE workers often work under gig-to-gig uncertainty, where goal-directed striving (the “will” and “way”; Snyder et al., 1996) may be particularly vital. By contrast, the structural burdens faced by business owners (e.g., regulations, financial risk) may dampen the motivational returns of hope (Eib and Bernhard-Oettel, 2024; Eib and Weidenstedt, 2025; Stephan et al., 2023).

Future research should further investigate PsyCap facets separately, use longitudinal and person-centered designs (Bergman and Lundh, 2015). In addition, more in-depth studies concerning the role of psychological capital factors may be warranted. Prior findings highlight that building metacognitive awareness could be valuable in developing hope and self-efficacy (Kleka et al., 2024), and since professional development in SSE is mainly self-directed (Cropanzano et al., 2022), better insight into how such awareness can be fostered would be of relevance.

The hypothesized curvilinear association between risk tolerance and job as well as life satisfaction was not supported, diverging from economic studies linking extreme risk attitudes to reduced entrepreneurial success (Caliendo et al., 2010, 2014; Kritikos, 2022). The discrepancy may reflect differences in outcomes (objective business survival vs. subjective job and life satisfaction) and measurement approaches. Still, risk tolerance showed a negative association with life satisfaction among business owners while statistically adjusting for age and PsyCap variables, possibly due to accumulated resource losses absorbed independently (Hobfoll, 2002). We also found that business owners reported higher risk tolerance than solo self-employed non-business owners. The results may, at least in part, illustrate a selection effect on the labor market: Individuals who are not very risk tolerant may prefer working in SSE as non-business owners, so that they can externalize administrative risks to umbrella companies. A recent interview study illustrates this viewpoint, as several solo self-employed working through umbrella companies stressed the low risk and their happiness about being able to outsource all administrative burdens related to difficult tax declarations, clients not paying etc., to umbrella companies (Müller et al., 2025). In such an SSE context, risk tolerance does not significantly associate with life satisfaction. It may thus not be surprising that a statistically significant risk tolerance-life satisfaction link was found only in solo self-employed business owners, whose SSE context may be slightly closer to an entrepreneurial one, so that risk tolerance, as part of an entrepreneurial personality, plays a larger role (Howard and Boudreaux, 2024). The fact that higher levels of risk tolerance were associated with lower levels of life satisfaction in a linear manner raises a number of questions for future studies, and may offer a nuanced explanation for the “mixed findings” previously observed in the literature (Binder and Blankenberg, 2021), suggesting that high risk-taking may exacerbate the erosion of life satisfaction often attributed to blurred work-life boundaries (Stephan, 2018). One such question may be whether this linear and negative link is be related to the context of solo businesses, which perhaps are too small to harbor, and profit from, risky investments and have more difficulties to absorb economic shocks. Here, it may also be interesting to disentangle the meaning of risk tolerance, self-efficacy, and overconfidence, since overconfidence has been found to relate to business failures in solo self-employment (Cieślik et al., 2023). If so, this would theoretically mean that low levels of risk tolerance, and thus, cautious investments, are a personal resource of advantage for SSE business owners. Another question is how the observed significant relation plays out over time; based on COR (Hobfoll, 1989) it is conceivable that loss spirals develop so that lower life satisfaction perhaps also triggers more risky decisions, as a means to compensate for earlier losses. Yet another question is whether linking risk tolerance to wellbeing – as done in this study – is what explains the absence of a significant curvilinear effect. Even if entrepreneurial success is likely to regain momentum towards the high end of risk tolerance, our results may signal that this is not the case for general life satisfaction, since perhaps stress and worries related to high risk taking may not be compensated for by successful business results alone. All these questions warrant more investigation to better understand in what way risk tolerance is an important personal resource (or risk) factor (Howard and Boudreaux, 2024) and what it means for business results and wellbeing in SSE as compared to larger business context.

Furthermore, our findings need to be interpreted within Sweden’s robust welfare context. A strong social safety net mitigates the financial risks of business failure, potentially explaining why risk tolerance did not positively relate to job or life satisfaction in our sample. This contrasts with regions like Latin America, Africa, or Asia, where self-employment is often necessity-driven due to weaker safety nets (e.g., Baluku et al., 2021). In such environments, risk tolerance and resilience may be far more critical for wellbeing. Consequently, future research should investigate these personal resources across more diverse geographical and cultural contexts. Also, solo self-employment as non-business owner may be a rather paradox construction that situates these individuals in between employment and self-employment (Hedenus and Nergaard, 2021; Müller et al., 2025). However, in other countries intermediate categories exist such as wage-portage in France and Belgium (Beuker and Pichault, 2022) or the so-called non-employed employees in Norway (Hedenus and Nergaard, 2021). Our results suggest that psychological capital and risk tolerance relate in similar ways to job and life satisfaction for the solo self-employed in Sweden, regardless of whether they are business owners or not. Whether results are similar for comparable intermediate categories in other labor market contexts still needs to be studied in order to better understand the heterogeneity of solo self-employment and its differences and similarities to gig employment (Watson et al., 2021).

## Strengths and limitations

This study is one of the few that singles out solo self-employment, a growing form of employment (Cieślik et al., 2025; van der Zwan et al., 2020), for which conditions and consequences need to be better understood. In addition, this study extends the understanding of solo self-employment as an employment that can include business and non-business owners. The latter group, non-owners, who are employed during the time of their assignments by an umbrella company, share features with both employment and self-employment (Hedenus and Nergaard, 2021). Legally, this group can be considered as short-term temporary employment or gig-employment (Bernhard-Oettel et al., 2025), while at the same time, workers themselves rather associate their way of working with that of self-employment (Müller et al., 2025). A comparative study as this adds to the understanding of similarities and dissimilarities, thereby facilitating the positioning of this group vis-à-vis employment and self-employment. Another strength of this study is the collection of quantitative data by means of questionnaires, making more in-depth statistical analyses possible. This advances knowledge particularly for the group of non-business owners, which hereto have been studied scarcely, and often with small-scale cases (Hedenus and Nergaard, 2021).

As with all research, however, there are also certain limitations to this study that deserve a short discussion. A first limitation concerns the cross-sectional design of the study, hampering the study of longitudinal, over-time effects, so that the direction of the found relationships, and potential loss or gain spirals (Hobfoll, 1989) could not be analyzed. Another limitation concerns the shortened measures used for psychological capital. The number of items used to measure hope and resilience was underrepresented in this study in comparison to the original scale developed by Luthans et al. (2007), which may have resulted in a lack of sufficient variance and suboptimal model fit. In addition, single-item measures were used for job and life satisfaction, to prevent survey fatigue and low completion rates. However, single-item measures have been found to perform very similarly to multiple-item measures (Cheung and Lucas, 2014) and the same has been found for many organizational and job-related variables (Matthews et al., 2022). A final limitation may concern sample representativeness. Our sample has a relatively high age and education. However, there is evidence that solo self-employed workers have higher educational attainment (van der Zwan and Hessels, 2019), and the age range fits well with the majority of Swedish self-employed workers, who are 45 years or older (Statistics Sweden, 2023). Representativeness is perhaps most difficult to estimate when it comes to the SSE non-business owners, since this group has fairly been studied before.

## Conclusion and practical implications

Ultimately, this study suggests that job and life satisfaction in solo self-employment is a matter of hope and optimism rather than tolerance for risks. While structural distinctions between independent business owners and umbrella-contracted workers exist, our findings suggest a shared psychological reality: future-oriented cognitions, specifically hope and optimism, serve as vital resources that could sustain wellbeing. Furthermore, contrary to the “traditional” entrepreneurial narrative, high risk tolerance does not inherently lead to a happier life; for SSE business owners, it may even act as a vulnerability factor. However, more research is needed to draw any firm conclusions, especially in regard to how these studied personal resources interact and build on each other over time. With that in mind, our data do suggest that practitioners and policymakers aiming to support the growing SSE workforce should look beyond financial safety nets and skills training, and also offer interventions that foster the psychological agency to visualize and plan for a positive and less risky future. This may help to ensure that the freedom of solo self-employment results in satisfaction with work and life in general.

## Data Availability

The raw data supporting the conclusions of this article will be made available by the authors, without undue reservation.

## Footnotes

1 While we posit that these theoretical associations apply to solo self-employment generally, we conducted the empirical analyses separately for business owners and non-business owners. This multi-group approach was necessary to explicitly test the assumption of similarity across employment structures, as addressed in our subsequent research questions.

2 Copyright © 2007 Fred L. Luthans, Bruce J. Avolio, and James B. Avey. All rights reserved in all media. Published by Mind Garden, Inc., www.mindgarden.com
