# Supplementary material for: Associations between psychological capital, risk tolerance, and satisfaction: a study of Swedish solo self-employed workers
Source: Front Psychol. 2026 Apr 16;17:1780099. doi: 10.3389/fpsyg.2026.1780099 (PMC13134391; doi:10.3389/fpsyg.2026.1780099)
Supplement: Supplementary file 1 [file Table_1.docx]

Supplementary Material

## Confirmatory Factor Analyses

Confirmatory factor analyses (CFAs) were employed for each sample to ensure the scale's factor structure using robust full information maximum likelihood estimation. Four models were compared: a single factor (1; all items loaded into a single factor), a four-factor (2; self-efficacy, hope, optimism & resilience), and a second-order structure (3; the 4 factors load into a higher-order factor of overall PsyCap), a bifactor model (including all 4 factors in addition to a general factor for PsyCap). However, estimating the fourth bifactor model led to convergence issues and was therefore excluded. A scaled chi-squared difference test (Satorra & Bentler, 2001) and change in fit indices were examined. The first model (all items loaded into a single factor) did not fit the data particularly well for either business owners (χ² [77] = 576.070, p <.001, CFI = .767, RMSEA = .140, SRMR = .075) or non-business owners (χ² [77] = 388.657, p <.001, CFI = .723, RMSEA = .161, SRMR = .090). Among business owners (N = 451), both the four-factor model (χ² [71] = 207.999, p <.001, CFI = .939, RMSEA = .075, SRMR = .054) and the second order model (χ² [73] = 211.934, p <.001, CFI = .938, RMSEA = .074, SRMR = .054) showed a moderate fit to the data, with only minor differences in fit indices (Δχ² [2] = 3.876, p = .144, ΔCFI = .001, ΔRMSEA = .000, ΔSRMR = .001). Standardized factor loadings ranged from .52 to .90 when using a four-factor solution. Factor intercorrelations ranged between .57 and .70.

For non-business owners (N = 225), the four-factor model showed moderate fit indices (χ² [71] = 175.797, p <.001, CFI = .917, RMSEA = .092, SRMR = .079), with a slightly high RMSEA value. However, the third model (χ² [73] = 182.871, p <.001, CFI = .912, RMSEA = .093, SRMR = .080) did show a slightly worse fit in comparison to model 2

(Δχ² [2] = 6.616, p = .037, ΔCFI = -.005, ΔRMSEA = .001, ΔSRMR = .001). The four-factor model was therefore preferred in this sample. Standardized factor loadings ranged from .43 to .93. Factor intercorrelations ranged between .48 and .73.


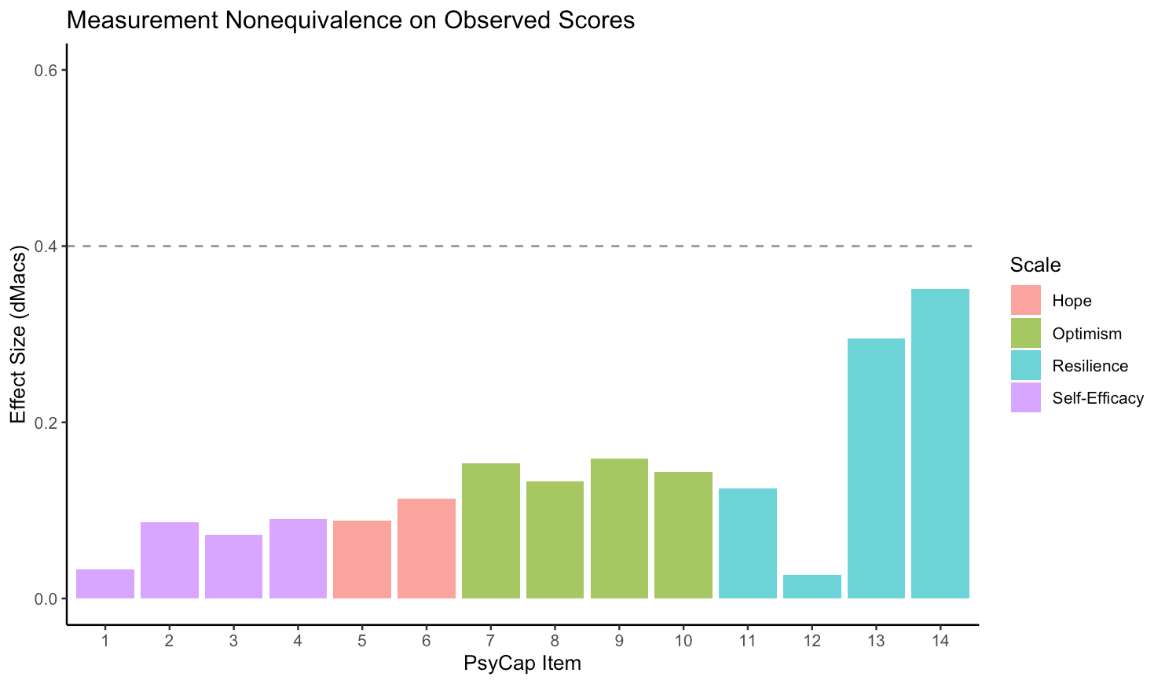


**Supplementary Figure 1**. Measurement nonequivalence (dMacs) on observed PsyCap scores (Multi-group CFA, 4-factors). The dashed line represents a small statistical effect size.

## Supplementary Tables

| **Supplementary Table 1**. Freely estimated regression coefficients across groups with Job Satisfaction as outcome (MGSEM), not including PsyCap. | | | | | | | | | |
| --- | --- | --- | --- | --- | --- | --- | --- | --- | --- |
|  | Business owners (*N* =454) | | |  |  | Non-business owners (*N*=222) | | |  |
|  | *b* | (CI95) | *β* | *p* |  | *b* | (CI95) | *β* | *p* |
| Risk Tolerance | -.35 | (-.79, .09) | -.09 | .119 |  | -.23 | (-1.02, .57) | -.04 | .578 |
| Risk Tolerance^2 | 1.63 | (.54, 2.72) | .15 | .003 |  | -1.62 | (-4.10, .86) | -.09 | .201 |
| Gender [Man] | -.14 | (-.33, .06) | -.07 | .163 |  | .08 | (-.23, .39) | .03 | .622 |
| Age | .02 | (.01, .02) | .19 | <.001 |  | .02 | (.00, .03) | .18 | .008 |
| Tenure | .00 | (-.01, .01) | -.01 | .772 |  | -.01 | (-.04, .02) | -.05 | .549 |
| Education [High] | -.18 | (-.36, .01) | -.09 | .060 |  | -.20 | (-.51, .10) | -.09 | .192 |
| ***R****^2^* |  | | | .06 |  |  | | | .06 |
| *Note*. CI95 represents the 95% confidence interval for the unstandardized coefficient (*b*). *β =* Standardized coefficient.  Model fit: χ² (366) = 1199.521, *p* < .001, CFI = .826, RMSEA = .089, SRMR = .127.  **p* <.05, ***p* <.01, ****p* <.001 | | | | | | | | | |
| **Supplementary Table 2**. Freely estimated regression coefficients across groups with Life Satisfaction as outcome (MGSEM), not including PsyCap. | | | | | | | | | |
|  | Business owners (*N*=454) | | |  |  | Non-business owners (*N*=222) | | |  |
|  | *b* | (CI95) | *β* | *p* |  | *b* | (CI95) | *β* | *p* |
| Risk Tolerance | -.35 | (-.75, ,05) | -.10 | .086 |  | -.19 | (-.86, .49) | -.04 | .584 |
| Risk Tolerance^2 | .99 | (-.11, 2.08) | .10 | .077 |  | .10 | (-1.88, 2.08) | .01 | .923 |
| Gender [Man] | .01 | (-.17, .19) | .00 | .928 |  | .11 | (-.15, .36) | .05 | .417 |
| Age | .01 | (.01, .02) | .19 | <.001 |  | .02 | (.01, .03) | .27 | <.001 |
| Tenure | .00 | (-.01, .01) | -.03 | .617 |  | -.02 | (-.04, -.00) | -.12 | .049 |
| Education [High] | .00 | (-.17, .17) | .00 | .995 |  | .14 | (-.10, .39) | .08 | .253 |
| ***R****^2^* |  | | | .04 |  |  | | | .09 |
| *Note*. CI95 represents the 95% confidence interval for the unstandardized coefficient (*b*). *β =* Standardized coefficient.  Model fit: χ² (366) = 1199.521, *p* < .001, CFI = .826, RMSEA = .089, SRMR = .127.  **p* <.05, ***p* <.01, ****p* <.001 | | | | | | | | | |

| **Supplementary Table 3**. Freely estimated regression coefficients across groups with Job Satisfaction as outcome (MGSEM), including all variables. | | | | | | | | | |
| --- | --- | --- | --- | --- | --- | --- | --- | --- | --- |
|  | Business owners (*N* = 457) | | |  |  | Non-business owners (*N*= 228) | | |  |
|  | *b* | (CI95) | *β* | Δ***R***^2^ |  | *b* | (CI95) | *β* | Δ***R***^2^ |
| Hope | .46*** | (.30, .62) | .51 | .13 |  | .36** | (.08, .64) | .38 | .06 |
| Self-Efficacy | -.40** | (-.69, -.10) | -.25 | .03 |  | .05 | (-.42, .52) | .03 | .00 |
| Resilience | .23 | (-.14, .60) | .11 | .01 |  | -.10 | (-.65, .45) | -.05 | .00 |
| Optimism | .18^a^ | (-.01, .37) | .16 | .01 |  | .29* | (.01, .56) | .25 | .03 |
| Risk Tolerance | -.20 | (-.60, .20) | -.05 | .00 |  | .09 | (-.59, .77) | .02 | .00 |
| Risk Tolerance^2 | .65 | (-.44, 1.73) | .06 | .00 |  | -1.55 | (-3.78, .68) | -.09 | .01 |
| Gender [Man] | -.19* | (-.36, -.01) | -.09 | .01 |  | .03 | (-.22, .27) | .01 | .00 |
| Age | .01* | (.00, .02) | .09 | .01 |  | .00 | (-.01, .01) | .05 | .00 |
| Tenure | .00 | (-.01, .01) | -.01 | .00 |  | .00 | (-.02, .02) | .00 | .00 |
| Education [High] | -.15 | (-.31, .02) | -.07 | .00 |  | -.12 | (-.39, .15) | -.05 | .01 |
| ***R****^2^* |  | | | .33 |  |  | | | 34 |
| *Note*. CI95 represents the 95% confidence interval for the unstandardized regression coefficient (*b*). *β =* Standardized regression coefficient.  Model fit: χ² (370) = 915.018, *p* < .001, CFI = .888, RMSEA = .071, SRMR = .075  ^a^ *p* = .060  **p* <.05, ***p* <.01, ****p* <.001 | | | | | | | | | |

| **Supplementary Table 4**. Freely estimated regression coefficients across groups with Life Satisfaction as outcome (MGSEM), including all variables. | | | | | | | | | |
| --- | --- | --- | --- | --- | --- | --- | --- | --- | --- |
|  | Business owners (*N* = 463) | | |  |  | Non-business owners (*N* =228) | | |  |
|  | *b* | (CI95) | *β* | Δ***R***^2^ |  | *b* | (CI95) | *β* | Δ***R***^2^ |
| Hope | .26*** | (.13, .39) | .32 | .05 |  | .49*** | (.29, .69) | .61 | .15 |
| Self-Efficacy | -.08 | (-.33, .18) | -.06 | .00 |  | -.32 | (-.74, .11) | -.22 | .02 |
| Resilience | -.23 | (-.56, .11) | -.12 | .01 |  | -.03 | (-.43, .37) | -.01 | .00 |
| Optimism | .44*** | (.27, .62) | .45 | .09 |  | .33** | (.12, .55) | .34 | .05 |
| Risk Tolerance | -.39* | (-.71, -.06) | -.11 | .01 |  | .18 | (-.34, .71) | .04 | .00 |
| Risk Tolerance^2 | .01 | (-.87, .89) | .00 | .00 |  | .11 | (-1.49, 1.72) | .01 | .00 |
| Gender [Man] | .02 | (-.13, .17) | .01 | .00 |  | .07 | (-.12, .26) | .04 | .00 |
| Age | .00 | (.00, .01) | .04 | .00 |  | .01 | (-.00, .02) | .12 | .01 |
| Tenure | .00 | (-.01, .01) | -.01 | .00 |  | -.01 | (-.03, .01) | -.06 | .00 |
| Education [High] | .05 | (-.10, .20) | .03 | .00 |  | .26** | (.08, .44) | .14 | .02 |
| ***R****^2^* |  | | | .34 |  |  | | | .54 |

*Note*. CI95 represents the 95% confidence interval for the unstandardized coefficient (*b*). *β =* Standardized coefficient.

Model fit: χ² (370) = 915.018, *p* < .001, CFI = .888, RMSEA = .071, SRMR = .075.

**p* <.05, ***p* <.01, ****p* <.001
